# Poly[μ-5-ammonio­isophthalato-aqua-μ-oxalato-dysprosium(III)]

**DOI:** 10.1107/S1600536809019199

**Published:** 2009-06-06

**Authors:** Liu-Shui Yan, De-He Huang, Chong-Bo Liu

**Affiliations:** a School of Environment and Chemical Engineering, Nanchang Hangkong University, Nanchang 330063, People’s Republic of China

## Abstract

The title complex, [Dy(C_8_H_6_NO_4_)(C_2_O_4_)(H_2_O)]_*n*_, is a dysprosium coordination polymer with mixed anions and was obtained under hydrothermal conditions. In the structure, the oxalate and 5-amino­isophthalate ligands link the dysprosium ions, building up a two-dimensional metal–organic framework parallel to the (10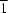) plane. These sheets are further connected through O—H⋯O, N—H⋯O and C—H⋯O hydrogen bonds, forming a three-dimensional supra­molecular structure.

## Related literature

For related structures, see: Chen *et al.* (2005 ▶); for isotypic structures, see: Liu *et al.* (2008 ▶).
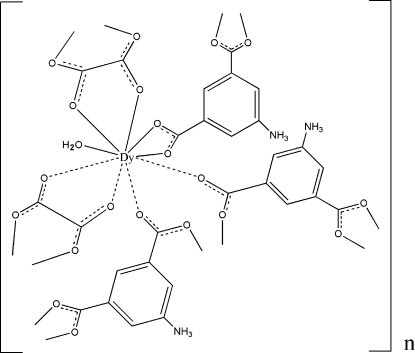


## Experimental

### 

#### Crystal data

[Dy(C_8_H_6_NO_4_)(C_2_O_4_)(H_2_O)]
*M* *_r_* = 448.67
Monoclinic, 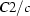

*a* = 19.951 (4) Å
*b* = 9.3967 (18) Å
*c* = 13.598 (3) Å
β = 118.478 (2)°
*V* = 2240.8 (8) Å^3^
*Z* = 8
Mo *K*α radiation
μ = 6.72 mm^−1^
*T* = 296 K
0.12 × 0.11 × 0.10 mm

#### Data collection

Bruker APEXII CCD diffractometer
Absorption correction: multi-scan (*SADABS*; Bruker, 2006 ▶) *T*_min_ = 0.499, *T*_max_ = 0.568 (expected range = 0.449–0.511)
8393 measured reflections
2089 independent reflections
1901 reflections with *I* > 2σ(*I*)
*R*_int_ = 0.109

#### Refinement

*R*[*F* ^2^ > 2σ(*F* ^2^)] = 0.026
*wR*(*F* ^2^) = 0.063
*S* = 1.08
2089 reflections
191 parameters
H-atom parameters constrained
Δρ_max_ = 1.29 e Å^−3^
Δρ_min_ = −1.56 e Å^−3^

### 

Data collection: *APEX2* (Bruker, 2006 ▶); cell refinement: *SAINT* (Bruker, 2006 ▶); data reduction: *SAINT*; program(s) used to solve structure: *SHELXS97* (Sheldrick, 2008 ▶); program(s) used to refine structure: *SHELXL97* (Sheldrick, 2008 ▶); molecular graphics: *PLATON* (Spek, 2009 ▶) and *XP* in *SHELXTL* (Sheldrick, 2008 ▶); software used to prepare material for publication: *SHELXL97*.

## Supplementary Material

Crystal structure: contains datablocks global, I. DOI: 10.1107/S1600536809019199/dn2450sup1.cif

Structure factors: contains datablocks I. DOI: 10.1107/S1600536809019199/dn2450Isup2.hkl

Additional supplementary materials:  crystallographic information; 3D view; checkCIF report

## Figures and Tables

**Table 1:** Hydrogen-bond geometry (Å, °)

*D*—H⋯*A*	*D*—H	H⋯*A*	*D*⋯*A*	*D*—H⋯*A*
O9—H2*W*⋯O3^i^	0.83	2.32	2.858 (4)	123
O9—H1*W*⋯O1^ii^	0.83	1.97	2.790 (4)	168
N1—H1*B*⋯O6^ii^	0.89	2.63	3.379 (5)	142
N1—H1*A*⋯O8^ii^	0.89	2.39	2.824 (5)	111
N1—H1*A*⋯O5^iii^	0.89	1.99	2.840 (5)	160
N1—H1*C*⋯O7^iv^	0.89	1.92	2.796 (6)	169
C2—H2⋯O9^ii^	0.93	2.55	3.421 (5)	157
C4—H4⋯O5^iv^	0.93	2.53	3.169 (6)	126

Symmetry codes: (i) 
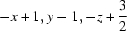
; (ii) 
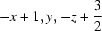
; (iii) 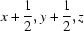; (iv) 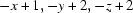.

**Fig. 1.**
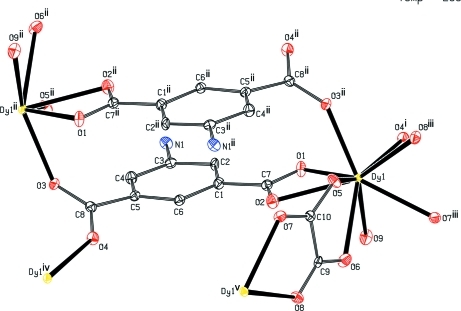
Coordination environment of the Dy(III) ion with the atom labeling scheme. Ellipsoids are drawn at the 30% probability level. H atoms have been omitted for clarity. [Symmetry codes: (i) x, y-1, z; (ii) -x+1, -y+2, -z+2; (iii) -x+1/2, y-1/2, -z+3/2; (iv) x, y+1, z; ]

**Fig. 2.**
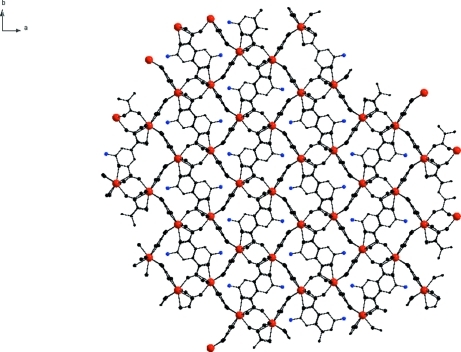
Packing view showing the 2-D metal organic framework. H atoms have been omitted for clarity.

## supplementary crystallographic information

### Comment

In recent years, the chemistry of supramolecular coordination polymers with mixed carboxylates has received much attention, and our group (Liu *et al.*, 2008) described the structure of europium and holmium coordination polymers with oxalate and 5-aminoisophthalate, the present dysprosium complex is similar to the europium and holmium complex.

In the title complex, the dysprosium ion is coordinated to nine oxygen atoms, among which one oxygen atom from one water molecule, four oxygen atoms from three HAPA ions, and the other four oxygen atoms from two oxalate ions. The two carboxylate groups of H_2_APA ligands are both completely deprotonated and exhibit chelating and bridging bidentate coordination modes respectively (Fig. 1). The amino group exist as –NH_3_^+^ (Chen *et al.*, 2005) . So, each HAPA ligand links three dysprosium atoms with Dy···Dy distances of 9.786, 9.397 and 5.419 Å, each oxalate ligand chelates two Dy(III) ions with a Dy···Dy distance of 6.259 Å, as shown in Fig. 1. The carboxylate groups of HAPA ligands link the Dy^3+^ ions to the dimeric units, which are further joined to a 2-D metal-organic framework containing regular parallelograms *via* HAPA ligands and OX ligands along *c* axis, as shown in Fig. 2. O—H···O and N—H···O hydrogen bonds link these layers to form a 3-D supramolecular structure.

The structure of the title complex is similar to that of other lanthanide (europium and holmium) coordination polymers with HAPA and oxalate ligands, and the mean Dy—O distance in the title complex of 2.430Å is between that of Eu—O (2.4728 Å) and Ho—O (2.4251 Å).

### Experimental

DyCl_3_.6H_2_O (0.038 g, 0.1 mmol), 0.018 g 5-aminoisophthalic acid (0.1 mmol), 0.013 g oxalic acid (0.1 mmol), 10 ml deionized water and 0.1 mmol 0.65 *M* NaOH aqueous solution were sealed in a 25 ml Teflon-lined stainless reactor and heated at 393 K for 72 h under autogeneous pressure, then cooled to room temperature. Colorless crystals of 1 were obtained. Anal. Calcd. for C10H8DyNO9 (448.67): C 26.75, H 1.78, N 3.12; found C 26.46, H 2.16, N 3.43.

### Refinement

The water H atoms were located in a difference Fourier map and refined with O—H distance restraints of 0.8287 and 0.8292 Å; all other H atoms were placed at geometrically idealized positions with C—H = 0.93 Å, N—H = 0.89 Å, and *U*_iso_(H) = 1.2 *U*_eq_(C,*N*).

### Crystal data

**Table:**

[Dy(C_8_H_6_NO_4_)(C_2_O_4_)(H_2_O)]	*F*(000) = 1704
*M**_r_* = 448.67	*D*_x_ = 2.660 Mg m^−^^3^
Monoclinic, *C*2/*c*	Mo *K*α radiation, λ = 0.71073 Å
Hall symbol: -C 2yc	Cell parameters from 5702 reflections
*a* = 19.951 (4) Å	θ = 2.5–28.2°
*b* = 9.3967 (18) Å	µ = 6.72 mm^−^^1^
*c* = 13.598 (3) Å	*T* = 296 K
β = 118.478 (2)°	Block, colourless
*V* = 2240.8 (8) Å^3^	0.12 × 0.11 × 0.10 mm
*Z* = 8	

### Data collection

**Table:**

Bruker APEXII CCD diffractometer	2089 independent reflections
Radiation source: fine-focus sealed tube	1901 reflections with *I* > 2σ(*I*)
graphite	*R*_int_ = 0.109
φ and ω scans	θ_max_ = 25.5°, θ_min_ = 2.3°
Absorption correction: multi-scan (*SADABS*; Bruker, 2006)	*h* = −24→24
*T*_min_ = 0.499, *T*_max_ = 0.568	*k* = −11→11
8393 measured reflections	*l* = −16→15

### Refinement

**Table:**

Refinement on *F*^2^	Primary atom site location: structure-invariant direct methods
Least-squares matrix: full	Secondary atom site location: difference Fourier map
*R*[*F*^2^ > 2σ(*F*^2^)] = 0.026	Hydrogen site location: inferred from neighbouring sites
*wR*(*F*^2^) = 0.063	H-atom parameters constrained
*S* = 1.08	*w* = 1/[σ^2^(*F*_o_^2^) + (0.0094*P*)^2^ + 0.8384*P*] where *P* = (*F*_o_^2^ + 2*F*_c_^2^)/3
2089 reflections	(Δ/σ)_max_ = 0.001
191 parameters	Δρ_max_ = 1.29 e Å^−^^3^
0 restraints	Δρ_min_ = −1.56 e Å^−^^3^

### Special details

**Table:**

Geometry. All e.s.d.'s (except the e.s.d. in the dihedral angle between two l.s. planes) are estimated using the full covariance matrix. The cell e.s.d.'s are taken into account individually in the estimation of e.s.d.'s in distances, angles and torsion angles; correlations between e.s.d.'s in cell parameters are only used when they are defined by crystal symmetry. An approximate (isotropic) treatment of cell e.s.d.'s is used for estimating e.s.d.'s involving l.s. planes.
Refinement. Refinement of *F*^2^ against ALL reflections. The weighted *R*-factor *wR* and goodness of fit *S* are based on *F*^2^, conventional *R*-factors *R* are based on *F*, with *F* set to zero for negative *F*^2^. The threshold expression of *F*^2^ > σ(*F*^2^) is used only for calculating *R*-factors(gt) *etc*. and is not relevant to the choice of reflections for refinement. *R*-factors based on *F*^2^ are statistically about twice as large as those based on *F*, and *R*- factors based on ALL data will be even larger.

### Fractional atomic coordinates and isotropic or equivalent isotropic displacement parameters (Å^2^)

**Table:**

	*x*	*y*	*z*	*U*_iso_*/*U*_eq_	
C1	0.5101 (3)	0.9321 (4)	0.8770 (5)	0.0176 (11)	
C2	0.5866 (3)	0.9047 (4)	0.9083 (4)	0.0168 (10)	
H2	0.6038	0.8119	0.9121	0.020*	
C3	0.6362 (2)	1.0187 (5)	0.9335 (4)	0.0161 (10)	
C4	0.6139 (3)	1.1553 (5)	0.9341 (4)	0.0198 (10)	
H4	0.6489	1.2293	0.9532	0.024*	
C5	0.5376 (2)	1.1837 (4)	0.9057 (4)	0.0159 (9)	
C6	0.4854 (3)	1.0707 (4)	0.8733 (4)	0.0154 (10)	
H6	0.4340	1.0889	0.8492	0.018*	
C7	0.4569 (3)	0.8075 (5)	0.8484 (4)	0.0170 (10)	
C8	0.5160 (3)	1.3320 (4)	0.9189 (4)	0.0171 (10)	
C9	0.2265 (2)	0.7844 (4)	0.6760 (4)	0.0143 (9)	
C10	0.2352 (2)	0.7786 (4)	0.7938 (4)	0.0142 (9)	
Dy1	0.367163 (11)	0.562032 (19)	0.814740 (18)	0.01134 (10)	
N1	0.7158 (2)	0.9903 (4)	0.9663 (4)	0.0198 (9)	
H1A	0.7278	1.0315	0.9178	0.030*	
H1B	0.7231	0.8968	0.9667	0.030*	
H1C	0.7452	1.0254	1.0344	0.030*	
O1	0.48363 (19)	0.6844 (3)	0.8541 (3)	0.0234 (8)	
O2	0.38806 (19)	0.8260 (3)	0.8218 (3)	0.0238 (8)	
O3	0.56915 (19)	1.4094 (3)	0.9915 (3)	0.0183 (7)	
O4	0.44868 (18)	1.3699 (3)	0.8570 (3)	0.0218 (8)	
O5	0.26983 (18)	0.6716 (3)	0.8522 (3)	0.0200 (7)	
O6	0.26011 (19)	0.6905 (3)	0.6506 (3)	0.0256 (8)	
O7	0.20849 (18)	0.8801 (3)	0.8235 (3)	0.0193 (7)	
O8	0.18745 (18)	0.8842 (3)	0.6150 (3)	0.0199 (7)	
O9	0.3929 (2)	0.5616 (3)	0.6572 (3)	0.0240 (8)	
H1W	0.4336	0.5912	0.6625	0.036*	
H2W	0.3713	0.5057	0.6041	0.036*	

### Atomic displacement parameters (Å^2^)

**Table:**

	*U*^11^	*U*^22^	*U*^33^	*U*^12^	*U*^13^	*U*^23^
C1	0.019 (3)	0.018 (2)	0.017 (3)	−0.0046 (17)	0.010 (2)	−0.0018 (18)
C2	0.020 (3)	0.0092 (18)	0.021 (3)	0.0011 (17)	0.009 (2)	−0.0042 (19)
C3	0.012 (2)	0.020 (2)	0.015 (3)	0.0006 (19)	0.005 (2)	0.000 (2)
C4	0.019 (2)	0.019 (2)	0.023 (3)	−0.0026 (19)	0.010 (2)	−0.001 (2)
C5	0.014 (2)	0.016 (2)	0.014 (2)	−0.0007 (18)	0.0033 (19)	−0.0011 (19)
C6	0.014 (2)	0.017 (2)	0.015 (3)	−0.0021 (17)	0.007 (2)	−0.0006 (18)
C7	0.021 (2)	0.017 (2)	0.014 (3)	−0.0022 (19)	0.009 (2)	−0.0014 (19)
C8	0.018 (2)	0.017 (2)	0.017 (3)	−0.0025 (19)	0.009 (2)	0.002 (2)
C9	0.011 (2)	0.013 (2)	0.017 (3)	−0.0031 (16)	0.005 (2)	−0.0055 (18)
C10	0.010 (2)	0.015 (2)	0.017 (3)	−0.0020 (16)	0.006 (2)	0.0007 (18)
Dy1	0.00982 (14)	0.00969 (13)	0.01363 (16)	−0.00032 (7)	0.00488 (12)	−0.00066 (7)
N1	0.017 (2)	0.0198 (19)	0.024 (3)	0.0041 (16)	0.011 (2)	0.0027 (18)
O1	0.0235 (17)	0.0157 (15)	0.033 (2)	−0.0055 (14)	0.0152 (17)	−0.0056 (16)
O2	0.0188 (16)	0.0216 (16)	0.031 (2)	−0.0024 (14)	0.0118 (15)	−0.0007 (15)
O3	0.0214 (18)	0.0152 (14)	0.016 (2)	−0.0012 (13)	0.0072 (16)	−0.0040 (14)
O4	0.0130 (17)	0.0173 (15)	0.027 (2)	0.0061 (13)	0.0032 (16)	−0.0008 (15)
O5	0.0158 (16)	0.0177 (15)	0.029 (2)	0.0055 (13)	0.0123 (15)	0.0086 (15)
O6	0.0208 (17)	0.0264 (17)	0.023 (2)	0.0051 (15)	0.0056 (16)	−0.0064 (16)
O7	0.0180 (17)	0.0205 (16)	0.017 (2)	0.0048 (13)	0.0069 (15)	−0.0020 (14)
O8	0.0205 (17)	0.0194 (16)	0.021 (2)	0.0051 (13)	0.0111 (16)	0.0044 (15)
O9	0.025 (2)	0.0261 (18)	0.029 (2)	−0.0066 (13)	0.0191 (19)	−0.0063 (14)

### Geometric parameters (Å, °)

**Table:**

C1—C6	1.385 (6)	C9—C10	1.528 (6)
C1—C2	1.399 (7)	C10—O7	1.250 (5)
C1—C7	1.502 (6)	C10—O5	1.263 (5)
C2—C3	1.386 (6)	Dy1—O4^i^	2.312 (3)
C2—H2	0.9300	Dy1—O3^ii^	2.332 (4)
C3—C4	1.359 (6)	Dy1—O1	2.413 (3)
C3—N1	1.455 (5)	Dy1—O8^iii^	2.426 (3)
C4—C5	1.408 (6)	Dy1—O9	2.429 (3)
C4—H4	0.9300	Dy1—O7^iii^	2.455 (3)
C5—C6	1.403 (6)	Dy1—O5	2.456 (3)
C5—C8	1.494 (6)	Dy1—O2	2.509 (3)
C6—H6	0.9300	Dy1—O6	2.541 (4)
C7—O2	1.254 (5)	N1—H1A	0.8900
C7—O1	1.260 (5)	N1—H1B	0.8900
C8—O4	1.249 (6)	N1—H1C	0.8900
C8—O3	1.277 (6)	O9—H1W	0.8287
C9—O8	1.248 (5)	O9—H2W	0.8292
C9—O6	1.252 (5)		
			
C6—C1—C2	120.2 (4)	O1—Dy1—O7^iii^	132.63 (11)
C6—C1—C7	121.9 (4)	O8^iii^—Dy1—O7^iii^	66.07 (11)
C2—C1—C7	118.0 (4)	O9—Dy1—O7^iii^	68.59 (11)
C3—C2—C1	118.7 (4)	O4^i^—Dy1—O5	143.90 (11)
C3—C2—H2	120.7	O3^ii^—Dy1—O5	77.07 (11)
C1—C2—H2	120.7	O1—Dy1—O5	121.80 (11)
C4—C3—C2	122.3 (4)	O8^iii^—Dy1—O5	70.08 (10)
C4—C3—N1	118.9 (4)	O9—Dy1—O5	134.73 (12)
C2—C3—N1	118.7 (4)	O7^iii^—Dy1—O5	101.09 (11)
C3—C4—C5	119.4 (4)	O4^i^—Dy1—O2	132.70 (11)
C3—C4—H4	120.3	O3^ii^—Dy1—O2	81.48 (11)
C5—C4—H4	120.3	O1—Dy1—O2	52.80 (10)
C6—C5—C4	119.1 (4)	O8^iii^—Dy1—O2	139.77 (10)
C6—C5—C8	122.0 (4)	O9—Dy1—O2	86.23 (10)
C4—C5—C8	118.7 (4)	O7^iii^—Dy1—O2	138.39 (11)
C1—C6—C5	120.1 (4)	O5—Dy1—O2	73.20 (10)
C1—C6—H6	119.9	O4^i^—Dy1—O6	141.86 (12)
C5—C6—H6	119.9	O3^ii^—Dy1—O6	135.64 (11)
O2—C7—O1	121.2 (4)	O1—Dy1—O6	106.59 (11)
O2—C7—C1	120.5 (4)	O8^iii^—Dy1—O6	109.04 (11)
O1—C7—C1	118.3 (4)	O9—Dy1—O6	70.64 (11)
O4—C8—O3	126.0 (4)	O7^iii^—Dy1—O6	72.89 (10)
O4—C8—C5	117.6 (4)	O5—Dy1—O6	64.31 (11)
O3—C8—C5	116.3 (4)	O2—Dy1—O6	67.58 (11)
O8—C9—O6	126.4 (5)	C3—N1—H1A	109.5
O8—C9—C10	116.5 (4)	C3—N1—H1B	109.5
O6—C9—C10	117.2 (4)	H1A—N1—H1B	109.5
O7—C10—O5	126.4 (4)	C3—N1—H1C	109.5
O7—C10—C9	117.3 (4)	H1A—N1—H1C	109.5
O5—C10—C9	116.2 (4)	H1B—N1—H1C	109.5
O4^i^—Dy1—O3^ii^	82.50 (12)	C7—O1—Dy1	95.1 (3)
O4^i^—Dy1—O1	80.12 (11)	C7—O2—Dy1	90.7 (3)
O3^ii^—Dy1—O1	75.29 (12)	C8—O3—Dy1^ii^	138.0 (3)
O4^i^—Dy1—O8^iii^	76.05 (11)	C8—O4—Dy1^iv^	142.5 (3)
O3^ii^—Dy1—O8^iii^	74.89 (11)	C10—O5—Dy1	116.9 (3)
O1—Dy1—O8^iii^	143.78 (12)	C9—O6—Dy1	115.4 (3)
O4^i^—Dy1—O9	78.37 (12)	C10—O7—Dy1^v^	119.0 (3)
O3^ii^—Dy1—O9	140.02 (12)	C9—O8—Dy1^v^	120.8 (3)
O1—Dy1—O9	67.07 (12)	Dy1—O9—H1W	122.3
O8^iii^—Dy1—O9	132.03 (11)	Dy1—O9—H2W	121.9
O4^i^—Dy1—O7^iii^	75.48 (11)	H1W—O9—H2W	111.7
O3^ii^—Dy1—O7^iii^	138.61 (10)		
			
C6—C1—C2—C3	−1.0 (8)	O4^i^—Dy1—O2—C7	4.5 (3)
C7—C1—C2—C3	179.1 (5)	O3^ii^—Dy1—O2—C7	76.0 (3)
C1—C2—C3—C4	3.3 (7)	O1—Dy1—O2—C7	−2.0 (3)
C1—C2—C3—N1	179.9 (4)	O8^iii^—Dy1—O2—C7	130.2 (3)
C2—C3—C4—C5	−1.6 (8)	O9—Dy1—O2—C7	−65.8 (3)
N1—C3—C4—C5	−178.2 (4)	O7^iii^—Dy1—O2—C7	−117.2 (3)
C3—C4—C5—C6	−2.3 (8)	O5—Dy1—O2—C7	155.0 (3)
C3—C4—C5—C8	173.4 (4)	O6—Dy1—O2—C7	−136.4 (3)
C2—C1—C6—C5	−2.9 (8)	O4—C8—O3—Dy1^ii^	103.1 (5)
C7—C1—C6—C5	177.0 (5)	C5—C8—O3—Dy1^ii^	−78.5 (5)
C4—C5—C6—C1	4.6 (8)	O3—C8—O4—Dy1^iv^	−0.3 (8)
C8—C5—C6—C1	−171.0 (5)	C5—C8—O4—Dy1^iv^	−178.6 (3)
C6—C1—C7—O2	−1.2 (8)	O7—C10—O5—Dy1	−149.7 (4)
C2—C1—C7—O2	178.7 (5)	C9—C10—O5—Dy1	29.4 (4)
C6—C1—C7—O1	−179.7 (5)	O4^i^—Dy1—O5—C10	−172.7 (3)
C2—C1—C7—O1	0.2 (7)	O3^ii^—Dy1—O5—C10	130.1 (3)
C6—C5—C8—O4	−31.5 (7)	O1—Dy1—O5—C10	66.8 (4)
C4—C5—C8—O4	152.8 (4)	O8^iii^—Dy1—O5—C10	−151.5 (3)
C6—C5—C8—O3	149.9 (4)	O9—Dy1—O5—C10	−21.4 (4)
C4—C5—C8—O3	−25.7 (6)	O7^iii^—Dy1—O5—C10	−92.2 (3)
O8—C9—C10—O7	−6.1 (5)	O2—Dy1—O5—C10	45.3 (3)
O6—C9—C10—O7	172.8 (4)	O6—Dy1—O5—C10	−27.5 (3)
O8—C9—C10—O5	174.8 (4)	O8—C9—O6—Dy1	160.0 (4)
O6—C9—C10—O5	−6.3 (5)	C10—C9—O6—Dy1	−18.8 (4)
O2—C7—O1—Dy1	−3.7 (5)	O4^i^—Dy1—O6—C9	170.5 (3)
C1—C7—O1—Dy1	174.8 (4)	O3^ii^—Dy1—O6—C9	−8.6 (4)
O4^i^—Dy1—O1—C7	−173.2 (3)	O1—Dy1—O6—C9	−94.4 (3)
O3^ii^—Dy1—O1—C7	−88.4 (3)	O8^iii^—Dy1—O6—C9	79.0 (3)
O8^iii^—Dy1—O1—C7	−123.9 (3)	O9—Dy1—O6—C9	−152.0 (3)
O9—Dy1—O1—C7	105.3 (3)	O7^iii^—Dy1—O6—C9	135.3 (3)
O7^iii^—Dy1—O1—C7	127.2 (3)	O5—Dy1—O6—C9	23.5 (3)
O5—Dy1—O1—C7	−24.2 (3)	O2—Dy1—O6—C9	−58.0 (3)
O2—Dy1—O1—C7	2.0 (3)	O5—C10—O7—Dy1^v^	−174.0 (3)
O6—Dy1—O1—C7	45.4 (3)	C9—C10—O7—Dy1^v^	7.0 (5)
O1—C7—O2—Dy1	3.5 (5)	O6—C9—O8—Dy1^v^	−176.7 (3)
C1—C7—O2—Dy1	−174.9 (4)	C10—C9—O8—Dy1^v^	2.1 (5)

Symmetry codes: (i) *x*, *y*−1, *z*; (ii) −*x*+1, −*y*+2, −*z*+2; (iii) −*x*+1/2, *y*−1/2, −*z*+3/2; (iv) *x*, *y*+1, *z*; (v) −*x*+1/2, *y*+1/2, −*z*+3/2.

### Hydrogen-bond geometry (Å, °)

**Table:**

*D*—H···*A*	*D*—H	H···*A*	*D*···*A*	*D*—H···*A*
O9—H2W···O3^vi^	0.83	2.32	2.858 (4)	123
O9—H1W···O1^vii^	0.83	1.97	2.790 (4)	168
N1—H1B···O6^vii^	0.89	2.63	3.379 (5)	142
N1—H1A···O8^vii^	0.89	2.39	2.824 (5)	111
N1—H1A···O5^viii^	0.89	1.99	2.840 (5)	160
N1—H1C···O7^ii^	0.89	1.92	2.796 (6)	169
C2—H2···O9^vii^	0.93	2.55	3.421 (5)	157
C4—H4···O5^ii^	0.93	2.53	3.169 (6)	126

Symmetry codes: (vi) −*x*+1, *y*−1, −*z*+3/2; (vii) −*x*+1, *y*, −*z*+3/2; (viii) *x*+1/2, *y*+1/2, *z*; (ii) −*x*+1, −*y*+2, −*z*+2.
